# Supplementary material for: Analysis of chi angle distributions in free amino acids via multiplet fitting of proton scalar couplings
Source: Magn Reson (Gott). 2024 Aug 19;5(2):103–20. doi: 10.5194/mr-5-103-2024 (PMC11570886; doi:10.5194/mr-5-103-2024)
Supplement: The supplement related to this article is available online at: https://doi.org/10.5194/mr-5-103-2024-supplement. [file mr-5-103-2024-supplement.zip › Syed2024/data/fit1d_fitnmr_output.tar.gz!/fit1d/arg/500/bmse000029_simulation_1_nmredata/1H/sparse_1d.pdf]

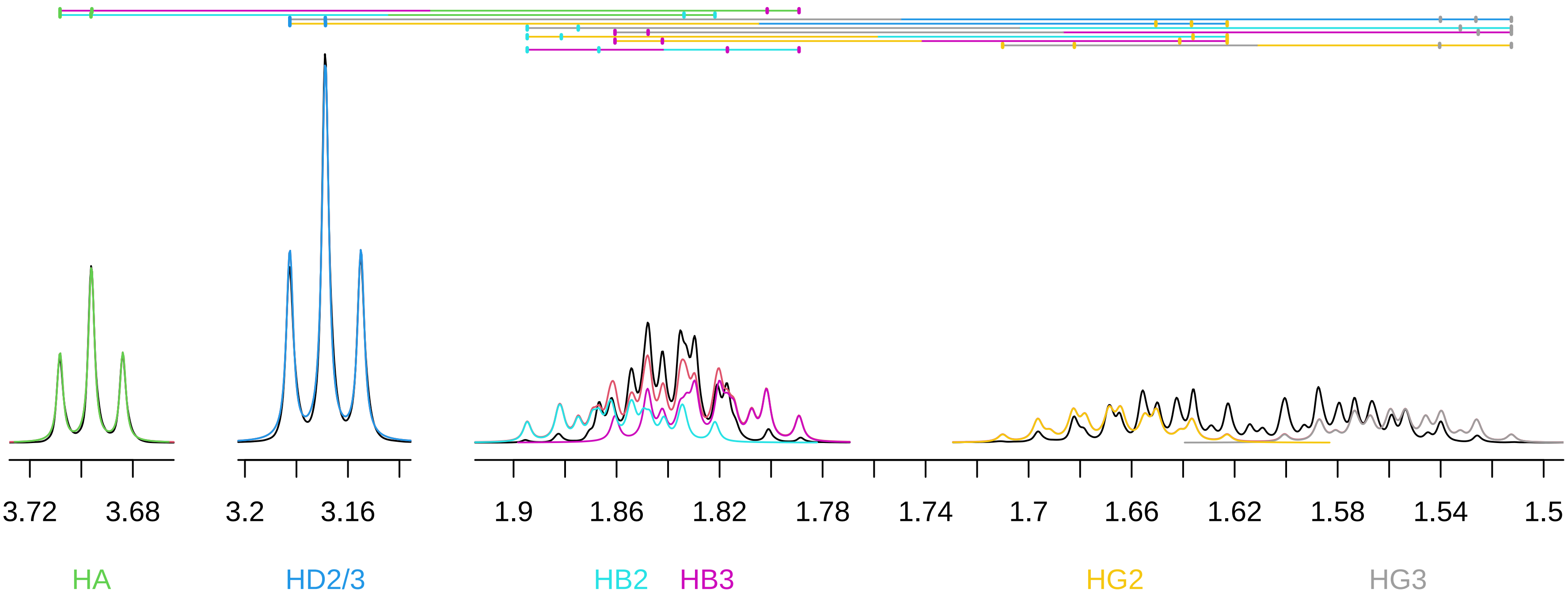
3.72
3.68
3.2
3.16
1.9
1.86
1.82
1.78
1.74
1.7
1.66
1.62
1.58
1.54
1.5
HA
HD2/3
HB2
HB3
HG2
HG3
